# Expression and Association of the *Yersinia pestis* Translocon Proteins, YopB and YopD, Are Facilitated by Nanolipoprotein Particles

**DOI:** 10.1371/journal.pone.0150166

**Published:** 2016-03-25

**Authors:** Matthew A. Coleman, Jenny A. Cappuccio, Craig D. Blanchette, Tingjuan Gao, Erin S. Arroyo, Angela K. Hinz, Feliza A. Bourguet, Brent Segelke, Paul D. Hoeprich, Thomas Huser, Ted A. Laurence, Vladimir L. Motin, Brett A. Chromy

**Affiliations:** 1 Lawrence Livermore National Laboratory, Livermore, CA, United States of America, 94550; 2 University of California Davis, NSF, Center for Biophotonics, Sacramento, CA, United States of America, 95817; 3 Humboldt State University, Department of Chemistry, Arcata, CA, United States of America, 95521; 4 University of Texas Medical Branch, Galveston, TX, United States of America, 77555; University of Helsinki, FINLAND

## Abstract

*Yersinia pestis* enters host cells and evades host defenses, in part, through interactions between *Yersinia pestis* proteins and host membranes. One such interaction is through the type III secretion system, which uses a highly conserved and ordered complex for *Yersinia pestis* outer membrane effector protein translocation called the injectisome. The portion of the injectisome that interacts directly with host cell membranes is referred to as the translocon. The translocon is believed to form a pore allowing effector molecules to enter host cells. To facilitate mechanistic studies of the translocon, we have developed a cell-free approach for expressing translocon pore proteins as a complex supported in a bilayer membrane mimetic nano-scaffold known as a nanolipoprotein particle (NLP) Initial results show cell-free expression of *Yersinia pestis* outer membrane proteins YopB and YopD was enhanced in the presence of liposomes. However, these complexes tended to aggregate and precipitate. With the addition of co-expressed (NLP) forming components, the YopB and/or YopD complex was rendered soluble, increasing the yield of protein for biophysical studies. Biophysical methods such as Atomic Force Microscopy and Fluorescence Correlation Spectroscopy were used to confirm that the soluble YopB/D complex was associated with NLPs. An interaction between the YopB/D complex and NLP was validated by immunoprecipitation. The YopB/D translocon complex embedded in a NLP provides a platform for protein interaction studies between pathogen and host proteins. These studies will help elucidate the poorly understood mechanism which enables this pathogen to inject effector proteins into host cells, thus evading host defenses.

## Introduction

In many gram-negative bacteria, host immune evasion is accomplished by injecting effector proteins into host cells using the type III secretion system (T3SS). [1,2] The main component of this system is a molecular syringe called the injectisome, which is capable of spanning the membranes of both the bacterium and the host organism. This complex is structurally and functionally conserved as an essential part of the mechanism for virulence in pathogens such as *Yersinia pestis (Y*. *pestis)*. Proteins involved in T3SS include cytotoxins and effectors (e.g. Yop proteins and Lcr-related proteins) that are secreted into eukaryotic cells and inhibit bacterial phagocytosis as well as modulate innate immunity. [1] However, the exact mechanism for how these proteins are actually deposited into cells from the bacteria is poorly understood.

For a number of organisms, the gross structural details of T3SS injectisomes have been revealed utilizing cryo-electron microscopy (EM) [3], providing substantial clues to the general architecture of the injectisome and the mechanism of protein translocation. However, cryo-EM studies of the *Y*. *pestis* injectisome have not been published. Despite the level of structural resolution gained from the aforementioned studies, novel techniques are needed to determine the stoichiometry and functional interactions of individual membrane-bound components of the injectisome. One of these membrane-bound complexes is believed to be responsible for maintaining contact between the injectisome and the host cell, through a channel inserted into the host plasma membrane. This injectisome complex has been coined the translocon [4, 5], and consists of proteins YopB, YopD and LcrV in *Yersinia*, each of which is required for delivery of effector proteins into the host cell [6]. LcrV has been shown by EM to form the tip of the T3SS injectisome pilus and is also required for insertion of YopB and YopD [7, 8, 9]. Insertion of YopB and YopD into the host cell membrane is a necessary step for entry of effector proteins such as YopJ into host cells. [7, 10]. However, YopD can also be isolated from the cytosol of the host cell and is involved in Yop regulation, secretion, and pore formation in *Yersinia pseudotuberculosis (Y*. *pseudotuberculosis*) [11, 12]. Recombinant *Y*. *pseudotuberculosis* YopB and YopD have previously been reported to interact and could be over expressed in modified systems [13, 14]. In addition, YopB could not be purified sufficiently for large scale biophysical studies [13]. The lack of an expression system for producing soluble full-length YopB or YopD alone or as part of complexes remains an impediment to biophysical characterization and structural analysis.

Therefore, the assembly of the translocon pore complex and interactions among the translocon pore proteins remain to be elucidated. Methods combining cell-free protein synthesis [15] with nanolipoprotein particle (NLP) technology [16] represent an ideal solution to express, isolate, and study protein expression and the functional complexes they form. Cell-free protein expression can be used to simultaneously co-express both a membrane protein and the apolipoprotein required for NLP formation in the presence of lipids [17]. The expressed components self-assemble into a nanometer scale sequestered membrane bilayer with embedded membrane protein and encircled by the amphipathic helix of the apolipoprotein [17]. The NLPs provide a unique environment within which membrane proteins may fold and form complexes. The resulting YopB-NLP complexes have been used in Fluorescence Correlation Spectroscopy (FCS) measurements to determine the binding constant of YopB binding to LcrV [18].

In this study, we demonstrate co-expression of soluble NLP proteins with the *Y*. *pestis* T3SS translocon proteins, YopB, YopD and YopB/D results in macromolecular NLP complexes containing the respective translocon proteins. Moreover, these results producing proteins inserted into NLPs further demonstrate the broad utility/application of cell-free expression for producing soluble and stable integral and associated membrane proteins as a complex, including proteins that have been difficult to express such as YopB.

## Results

**Fig 1:**
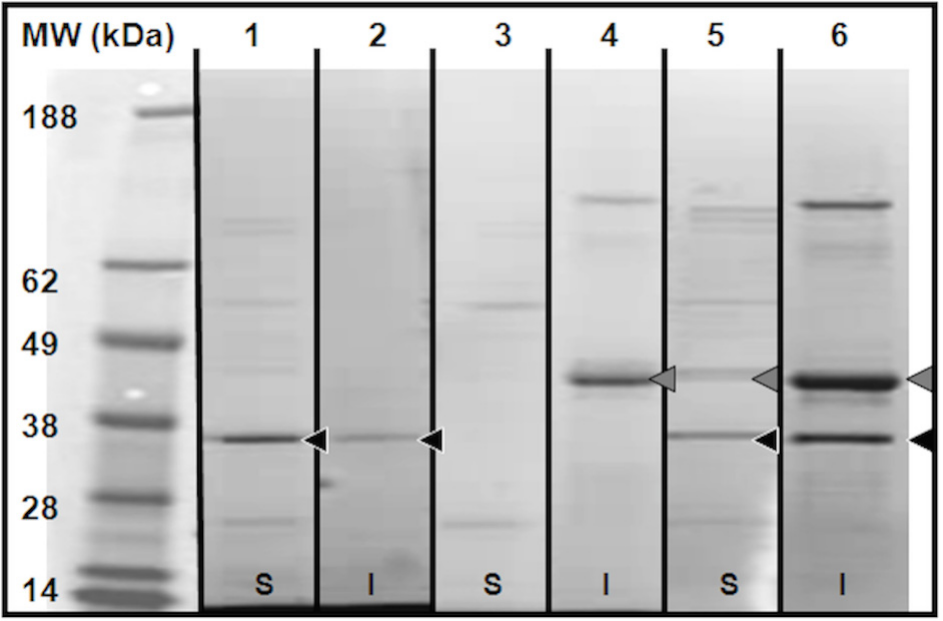
Soluble YopB is stabilized by YopD. Samples from cell-free expression of YopB, YopD and YopB/D were native affinity purified from cell-free expression. Lanes represent the following **1)** YopD elution; **2)** YopD precipitated on affinity beads; **3)** YopB elution; **4)** YopB precipitated on affinity beads; **5)** Elution of YopB co-expressed with YopD; **6)** YopB co-expressed with YopD precipitated on affinity beads. **Black** arrow indicated YopD, **Grey** arrow indicates YopB. Samples were run on a 4–12% Bis-Tris gel with MES-SDS running buffer and stained with Coomassie brilliant blue. Samples were run on a 4–12% Bis-Tris gel with MES-SDS running buffer and stained with Coomassie brilliant blue, followed by destaining and imaging. The molecular weight markers are SeeBlue Plus2 prestained markers from Life Technologies.

Cell-Free expressed YopB is stabilized when expressed with YopD. *Y*. *pestis* YopB has two proposed transmembrane domains [14, 19]. Proteins containing multiple transmembrane domains are often difficult to express and stabilize in their full length form. To avoid issues associated with cell-based expression of membrane proteins, such as protein precipitation, cytotoxicity and low yield as previously reported for membrane proteins or complex expression systems as required for YopB, [13] we used cell-free co-expression in an *E*. *coli* lysate expression system to produce recombinant His-tagged YopB and/or YopD [20]. Cell-free expression of YopD His-tagged followed by subsequent native immobilized metal Ni-affinity chromatography (IMAC) purification produced largely soluble YopD (Fig 1, lane 1). Expression and non-denaturing purification of YopB, on the other hand, produced insoluble material that was unable to be eluted from the Ni-affinity resin (Fig 1, lanes 3 & 4). The co-expression of YopB and YopD in the same cell-free reaction yielded some soluble YopB that could be purified (Fig 1, lanes 5 & 6), but a substantial portion remained in the insoluble fraction.

These results are similar to those reported previously with YopB and YopD from *Y*. *pseudotuberculosis* where YopB was stabilized in the presence of YopD and could be purified from cell-based overexpression. [12] Both YopB and YopD are highly conserved between the two *Yersinia* species, with > 99% sequence identity for each protein [19, 21]. These results suggest protein-protein interactions may have a stabilizing effect on YopB and YopD, e.g. possibly assisting in the correct folding of YopB by shielding the hydrophobic domains from aqueous environments. As judged by gel densitometry, following simultaneous expression of YopB and YopD in the absence of lipids, both proteins are solubilized to a greater degree as compared to when expressed individually (Table 1, YopB, individual: 14.1% together: 8.6%, and YopD, individual: 33.4%, together: 13.5%, respectively), suggesting a potential stabilizing interaction between both proteins.

**Table 1:** Percent of total Yop protein in the soluble fraction.

**Reactions expressing YopB**			**Reactions expressing YopD**		
**Reaction**	% soluble	stdev	**Reaction**	% soluble	stdev
YopB	14.1	4.5	YopD	33.4	20.3
YopB + lipid	15.2	4.7	YopD + Lipid	47.1	33.2
YopB-NLP	44	17.6	YopD-NLP	58.7	15.3
**Reactions expressing both YopB and YopD**					
**YopB Bands**	% soluble	stdev	**YopD Bands**	% soluble	stdev
YopB/D	8.6	1.5	YopB/D	13.5	0.6
YopB/D + Lipids	17.6	8	YopB/D + Lipids	17.1	6.9
YopB/D-NLP	28.3	8.8	YopB/D-NLP	41	8.9

**Fig 2:**
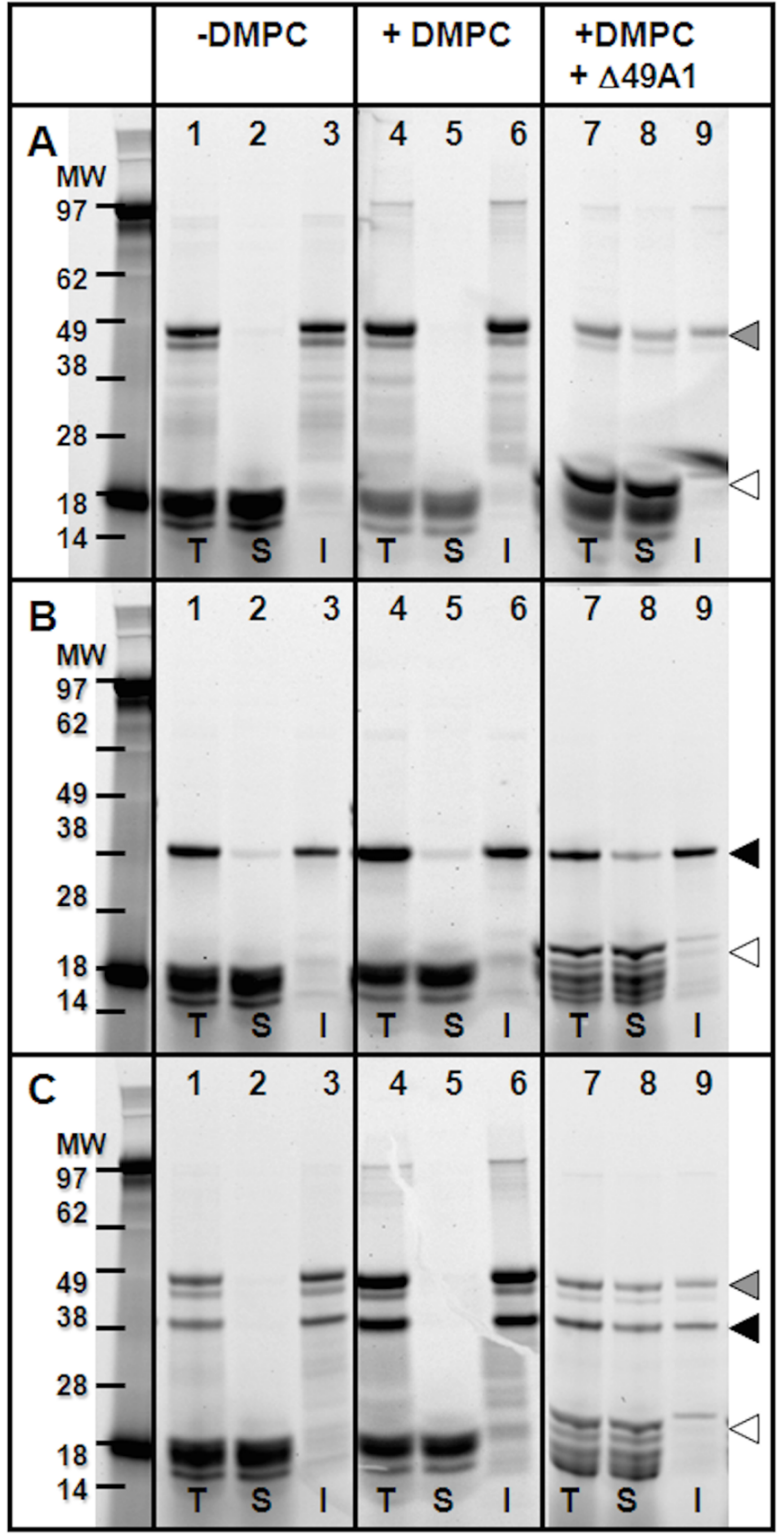
Co-expression of YopB, YopD, or YopB/D with NLP-forming Δ49ApoA1 increases the solubility of these proteins. SDS-PAGE of cell-free expressed **A)** YopB **B)** YopD and **C)** YopB/D. Lanes correspond to expression experiments **(Lanes 1–3)** in the absence of lipids; **(lanes 4–6)** in the presence of DMPC lipid vesicles; **(Lanes 7–9)** and in the presence of DMPC lipids as well as the co-expression of Δ49ApoA1. Crude reactions were separated into total **(T)**, soluble **(S)** and pellet **(I)** fractions, separated by a 4–12% Bis-Tris SDS-PAGE gel with MES-SDS running buffer. Proteins were detected by fluorescence of an incorporated Fluortect-green (Promega) labeled lysine added to the cell-free reactions. **Grey arrow** YopB; **Black arrow** YopD; **White arrow** Δ49ApoA1.

Cell free expression in the presence of lipids increases expression but not solubility of YopB and/or YopD. In an attempt to increase the yield and recovery of YopB and YopD, small unilamellar 1, 2-dimyristoyl-sn-glycero-3-phosphocholine (DMPC) vesicles were added to the cell-free reactions. The addition of lipids to cell free reactions has been previously utilized to increase the yield of membrane proteins [22]. DMPC was sonicated to optical clarity to produce liposomes and then added to the cell-free reaction mixture. Cell free expression of YopB and YopD together and individually in the presence of liposomes (Fig 2A–2C, lane 4) produced more protein as compared to reactions without the addition of lipids (Fig 2A–2C, lane 1). As measured by gel densitometry, YopB and YopD expressed alone in the presence of DMPC liposomes averaged a 1.21 fold increase as compared to expression without liposomes, and YopB and YopD expressed together averaged a 1.76 fold increase (Fig 2, lanes 1 vs. 4, (T) total).

In addition to the total yield, we compared the yield of YopB and YopD in the soluble fraction (S) for cell-free expression with liposomes. Individual expression of YopB in the presence of liposomes, produced mostly insoluble protein (P) and an increase in the percent of protein in the soluble fraction (15.2% as compared to 14.1% without liposomes, Table 1, Fig 2) as measured by densitometry gel analysis. Individual expression of YopD also showed an increase in percent solubility with the addition of liposomes (33.4→47.1%, Table 1, and Fig 2). Simultaneous expression of both proteins in the presence of lipid increased the percentage of soluble YopB and YopD compared to expression of YopB and YopD together without liposomes (YopB: 8.6% → 17.6%, YopD: 13.5% → 17.1%). These findings again suggest that a complex forms between YopB and YopD, in this case in the lipid environment, which may be indicative of the mechanism of translocon insertion into the host membrane. The hydrophobic nature of YopB could provide the driving force for complex formation at the tip of the injectisome that then intercalates into the host membrane.

Even if liposomes were able to provide high solubility for YopB or YopD protein expression, these complexes would still be less than ideal for characterizing the translocon complexes. These complexes are unstable and fail to provide access to both sides of a membrane protein within a single bilayer. In aqueous environments, liposomes rapidly coalesce into supramolecular lipid structures and this inherent instability limits the ability to characterize individual Yop translocon proteins or the complex formed by these proteins.

Cell free co-expression using NLPs, produces soluble YopB/D proteins. In an effort to increase the yield of soluble translocon complexes, we expressed YopB, YopD, and YopB/D using a cell-free NLP-based co-expression method [17]. By expressing these proteins as a complex using NLPs, we sought to enhance their study for protein-protein interactions and provide a bilayer structure that is likely similar to the environment found upon translocon pore formation in eukaryotic cells. YopB and/or YopD were co-expressed with the apolipoprotein Δ49ApoA1, an NLP forming scaffold protein, in the presence of a lipid involved in bulk eukaryotic bilayer formation. These reactions resulted in an increase in solubility of YopB and the YopB/D complex (Table 1, Fig 2). Co-expression resulted in substantial net-increases of percent solubility for the following: 44.0% YopB and 58.7% YopD when individually combined in NLPs, and 28.3% YopB and 41.0% YopD when together combined in NLPs, respectively, compared to their percent solubility when expressed individually or together with or without lipids (Table 1). The increased solubility demonstrates the ability of NLPs to improve the solubilization of YopB and YopB/D as compared to expression with liposomes or standard cell-free reactions. Interestingly, when YopD was expressed alone, there was only a slight increase in the solubility when including co-expression of the Δ49ApoA1 protein.

**Fig 3:**
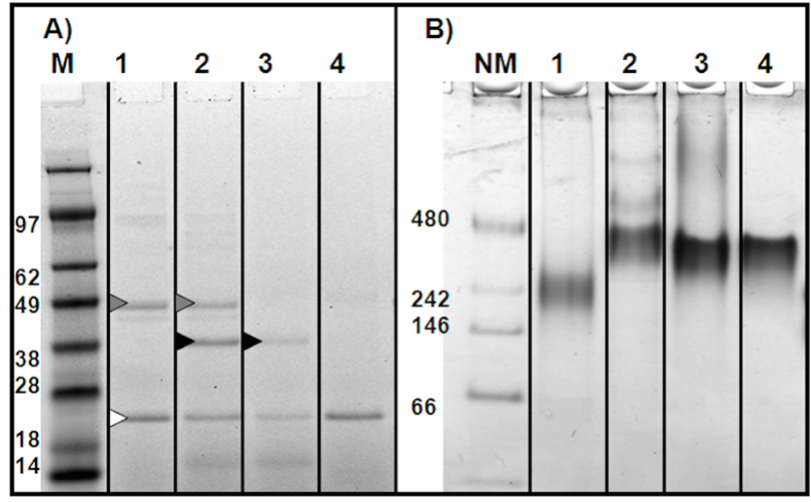
Gel electrophoresis indicates NLP complex formation with YopB and/or YopD. Purified expressed YopB and/or YopD co-expressed with Δ49ApoA1 in the presence of lipid. **A)** Denatured 4–12% Bis-Tris PAGE gel, MES-SDS buffer, Native Tris-glycine buffer, detection by SyproRuby fluorescence total protein stain. **B)** Native 4–12% Tris-glycine PAGE gel, Native Tris-glycine buffer, detection by SyproRuby fluorescence total protein stain. The lanes are represented as follows with the predicted molecular weight based on native gel size; **1)** YopB with Δ49ApoA1, 237 kDa complex; **2)** YopB/D with Δ49ApoA1, 448 kDa complex; **3)** YopD with Δ49ApoA1, 397 kDa complex; **4)** Δ49ApoA1 only (empty-NLP), 393 kDa complex; **M)** Mass standard (kDa) SeeBlue Plus 2 (Invitrogen); **NM)** Protein mass standard (kDa) NativeMark (Invitrogen). **Grey arrow** YopB; **Black arrow** YopD; **White arrow** Δ49ApoA1.

YopB and /or YopD are associated with NLPs as a soluble complex. In order to better characterize the complexes of YopB and/or YopD with discoidal NLPs, large scale (1 mL) cell-free reactions were prepared. The soluble fraction of these reactions were separated by centrifugation and purified using IMAC. These complexes were subjected to denatured and native gel electrophoresis (Fig 3). Denatured gel data show, YopB and/or YopD were purified along with Δ49A1 (Fig 3A). Native gel electrophoresis indicates NLP complex formation with YopB and YopD individually when expressed in the same reaction. Free YopB and YopD bands are absent in the native gel. For comparison, “empty”-Δ49A1-NLPs, encircling lipid alone, are shown (Fig 3A and 3B, lane 4) displaying the characteristic "empty" NLP band on the native gel [17]. YopB and/or YopD incorporation into NLPs are expected to show altered migration. Interestingly, the size of the YopB-NLP complex (~237 kDa) is smaller than the “empty”-NLP complex (~393 kDa), YopD-NLP complex (~397 kDa) and YopB/D-NLP complex (~441 kDa). Additionally, the YopB/D-NLP complex has two higher mass bands (1036 and 634 kDa), suggesting a structural alteration or the presence of additional oligomeric states in the formed complex.

The two proposed transmembrane domains in YopB may contribute to compact lipid packing in this NLP complex resulting in a faster migration in the native gel [14, 19]. YopD on the other hand possesses only one proposed transmembrane domain and one to two proposed amphipathic helices, allowing for less dense packing, hence larger particle size and slower migration. [12, 19] The YopB/D-NLP measured at 446 kDa by electrophoresis should accommodate 1–3 of each component assuming 4–6 Δ49ApoA1 scaffold proteins as previously modeled in a similar NLP with a defined number of lipids. The YopB/D-NLP measured at 441 kDa by electrophoresis should accommodate 1–3 of each component. Additionally the YopB/D-NLP complex has two higher mass bands (1036 and 634 kDa) which may represent larger NLP complexes with varying amounts of YopB and YopD incorporation and/or oligomerization.

**Fig 4:**
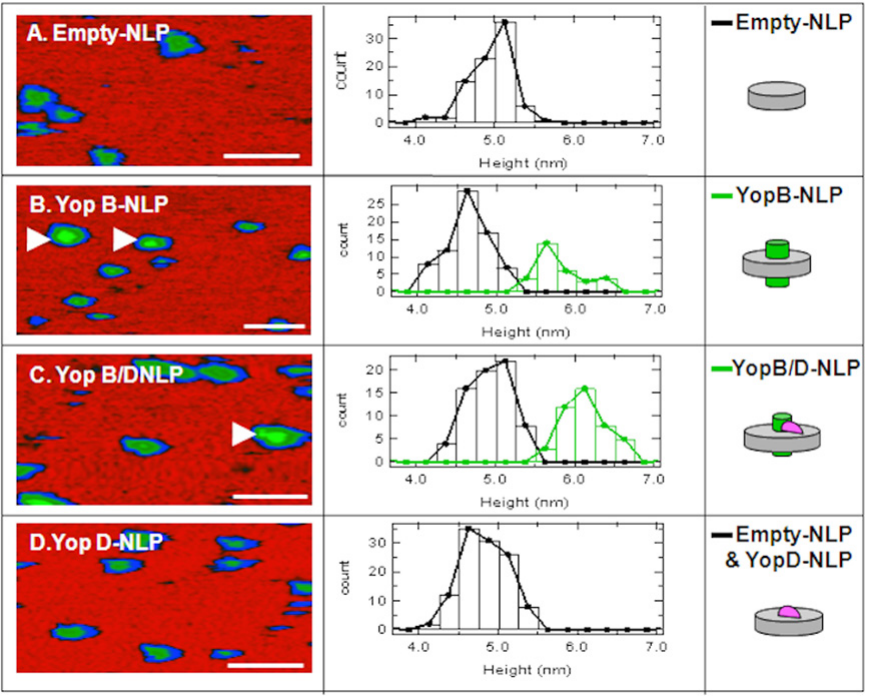
Atomic Force Microscopy (AFM) of empty- and Yop-NLPs. **Left panels**, Atomic force microscopy (AFM) image of Empty- and Yop-NLPs prepared by co-expression. 100 nm scale bar. **Center panels**, AFM height distribution plots. **Right panels**, NLP cartoons with and without Yop insertion. **A)** empty-NLPs. **B)** YopB-NLPs. **Black Line**: empty-NLPs representing 70% of the total; **Green Line**: co-expressed YopB-NLPs representing 30% of total. **C)** YopB/D-NLPs. **Black Line**: empty-NLPs representing 61% of the total; **Green Line**: co-expressed YopB-NLPs representing 39% of the total. **D)** YopD-NLPs. **Black Line** represents all particles; YopD-NLPs were indistinguishable from empty-NLPs.

Atomic Force Microscopy confirms YopB-NLP complex formation. Atomic force microscopy (AFM) was used to confirm that YopB and/or YopD can form a complex with NLPs. Previous studies with bacteriorhodopsin, a seven helix transmembrane protein, have shown that AFM can be used to determine the percent of membrane protein incorporation in lipid membranes and NLPs [17, 23–25]. YopB-NLPs, YopD-NLPs, YopB/D-NLPs and “empty”-NLPs were imaged on atomically flat mica. Representative images and the results of image analysis are shown in Fig 4 and Table 2. YopB-NLPs and YopB/D-NLPs showed populations with a significant height increase when compared to “empty”-NLPs (Fig 4, center panels and Table 2). NLPs incorporating YopB/D showed increased height as compared to "empty"-NLPs, 18.9 ± 4.9 vs. 14.5 ± 3 nm, respectively, consistent with higher order oligomerization states of YopB/D complexes. In addition to height measurements an incorporation rate of 30% for YopB and 39% for YopB/D was calculated (Table 2). The YopD-NLP complexes did not show a height increase by AFM (Table 2, and Fig 4).

**Table 2:** Size distribution and membrane protein incorporation rate of co-expressed YopB- and/or YopD-NLPs as determined by AFM.

Sample	Populations observed	Height (nm)	Diameter (nm)	Incorporation rate
Empty-NLPs	A: empty-NLPs only	4.6 ± 0.3	13.6 ±1.7	N/A
YopB-NLPs	B.1: empty-NLPs	4.6 ± 0.3	12.1 ± 2.1	---
	B.2: YopB-NLPs	5.8 ± 0.3*	14.0 ± 3.1	30%
YopB/D-NLPs	C.1: empty-NLPs	4.9 ± 0.3	14.5 ± 3.0	---
	C.2:YopB/D-NLPs	6.1 ± 0.3*	18.9 ± 4.8	39%
YopD-NLPs	D: empty- and YopD-NLPs	4.8 ± 0.3	14.9 ± 3.0	indeterminate

*p-values<0.001

YopB-NLP and YopB/D-NLP complex formation was confirmed by AFM (Fig 4B and 4C). Samples containing co-expressed YopB and YopB/D showed populations (30% and 39% respectively, Table 2) with increased height due to the presence of the Yop proteins (Fig 4B and 4C). Schematic images of the Yop proteins incorporated in the NLP disc are depicted in the right panels of Fig 4. These cartoons represent a portion of the height distribution plot (Fig 4, center panel, green line) showing the population of particles deviating from the height distribution profile of "empty" NLPs (Fig 4, center panels, black line), which is in agreement with previous work where a height increase was observed with the incorporation of the membrane protein bacteriorhodopsin [17, 22–26]. Of note is the large diameter measurement of the YopB/D-NLPs, which is, 18.9 ± 4.9 nm (Table 2). Possessing multiple transmembrane helices appears to affect how the protein protrudes from the zwitterionic lipid surface of the NLP bilayer as well as affecting native gel migration. YopB-NLPs are likely more compact, as observed by native PAGE migration (Fig 3) and by the AFM diameter measurements (Fig 4B, Table 2), with less DMPC in the bilayer, perturbing migration on the native gel.

In the sample containing co-expressed YopD (Fig 4D), height analysis by AFM could not distinguish NLPs with incorporated YopD from “empty”-NLPs containing only the lipid bilayer (Fig 4D, Table 2). Yet, denatured gel electrophoresis clearly shows a YopD band associated with this sample, (Fig 3A, lane 3) and native electrophoresis shows a characteristic NLP band. This may be consistent with the proposed structure of YopD, having only one proposed transmembrane region [27]. YopD also possesses one or two amphipathic domains [12, 28] near the c-terminus, which could interact at the periphery of the NLP disc similar to the amphipathic apolipoprotein or intercalate within the bilayer with the hydrophilic side of the helix exposed in proximity to the phospholipid head groups. The single transmembrane helix combined with two amphipathic helices intercalated in the membrane polar head groups of the NLP may not provide a height difference as compared to an "empty" DMPC bilayer. However, previous studies have shown that YopB and YopD will insert into artificial membranes when the pathogen is activated at 37°C and is in low Ca^+2^ environments. [29]

**Fig 5:**
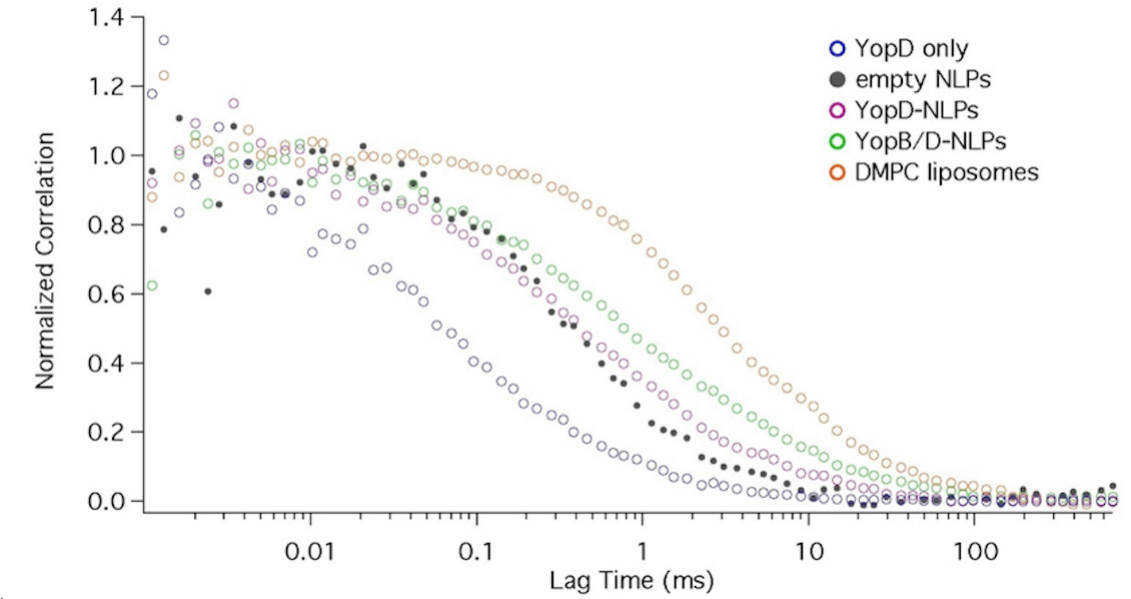
Diffusion curves of protein, empty NLPs, protein associated NLPs, and liposomes measured by FCS. The diffusion curves for YopD (**blue**) and the DMPC liposomes (**orange**) are derived from the autocorrelation of their corresponding fluorescence (YopD: bodipy, DMPC: Texas red) intensity time traces, while the diffusion curve for empty NLPs (**black**) is derived from the cross correlation of bodipy signal (labeled on Δ49ApoA1) and the Texas red signal (labeled on DMPC lipids) of their corresponding fluorescence intensity time traces, and the diffusion curves for YopD-NLPs (**purple**) and YopB/D-NLPs (**green**) are derived from the cross correlation of the GFP signal (labeled on Yop proteins) and the Texas red signal (labeled on DMPC lipids) of their corresponding fluorescence intensity time traces.

Fluorescence Correlation Spectroscopy reaffirms YopD- and YopB/D–NLP complex formation. Fluorescence correlation spectroscopy (FCS) was used to confirm YopD and YopB/D complex formation with NLPs and to help elucidate the structural differences among these complexes. FCS measurements of YopD-NLPs, YopB/D-NLPs and "empty" NLPs were performed on a MicroTime 200 single molecule fluorescence confocal microscope (PicoQuant, Berlin) with ~ 3-minute long measurements time traces. Correlation curves (Fig 5) were calculated using the manufacturer-provided SymPhoTime software. The diffusion time of each species was extracted by fitting the correlation curves using a three dimensional diffusion model:

G(τ)=G011+ττD11+κ2ττD

Where *G(t)* is the correlation function, *t* is the lag time, *G*_*0*_ is the correlation at zero lag time, *τ* is the diffusion time, and *κ* is the eccentricity of the effective volume. *κ* was calibrated and then fixed at 1.76 ± 0.02 for modeling. The instrument was calibrated with size standards, so the diffusion times of all the samples were converted to effective diameters [30]. Fitted diffusion times are shown in Table 3. For YopD-NLPs, the cross correlation (between Texas Red labeled lipids and GFP labeled YopD) confirmed that YopD was associated with the NLP. Compared to the diameter of YopD, the size of the complex was significantly larger, similar to that of "empty" NLPs, For YopB/D-NLPs, there was again a cross correlation between Texas Red signal (labeled lipids) and GFP signal (labeled YopB and YopD), confirming their association (Fig 5). Additionally, the correlation curve for YopB and YopD has a shallower slope, and did not fitted as well to the model. This suggests variability in the species size, consistent with the larger size observed earlier by AFM, and the slower mobility observed using gel electrophoresis. Moreover, YopB/D-NLPs showed a significant increase in size compared with YopD-NLPs or "empty" NLPs. A FCS measures molecular dynamics in the solution phase, combined with the AFM results, it supports the claim of YopB/YopD incorporation with NLPs in solution in the presence and absence of a surface.

**Table 3:** Size distribution for co-expressed YopD- and/or YopB/D-NLPs as determined by FCS.

Sample	Diffusion time (ms)	Diameter (nm)
Empty NLPs	0.51 ± 0.17	10.32 ± 4.75
YopD only	0.085 ± 0.011	3.57 ± 2.54
YopD-NLPs	0.60 ± 0.14	11.75 ± 4.20
YopB/D-NLPs	1.26 ± 0.18	22.22 ± 4.34
Liposomes	4.20 ± 0.58	68.89 ± 8.47

**Fig 6:**
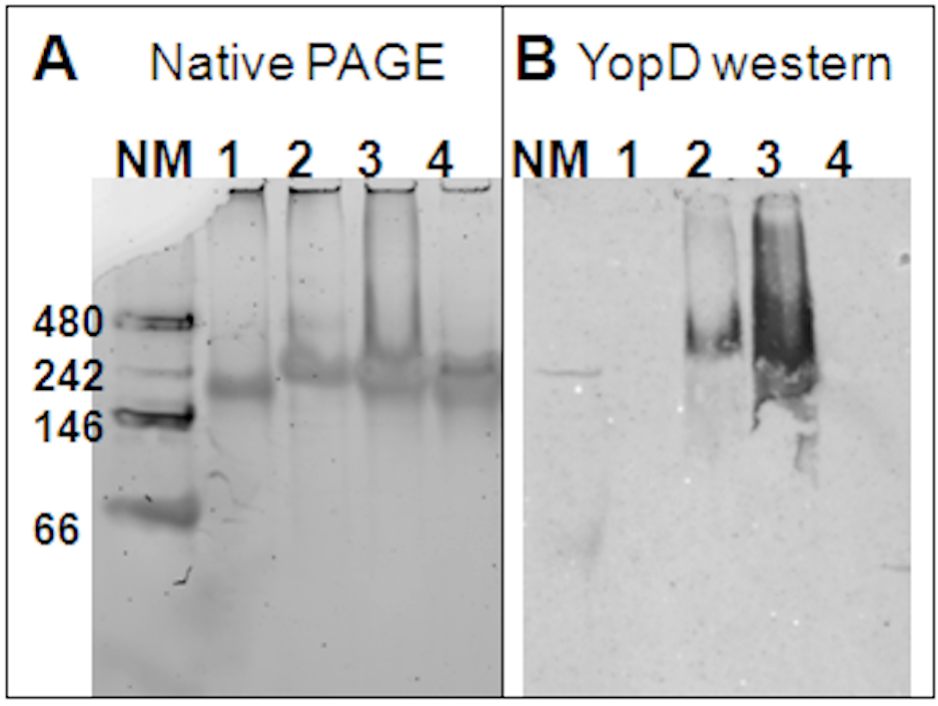
Anti-YopD western blot confirms YopD complex formation with NLPs. Purified expressed YopB and/or YopD co-expressed with Δ49ApoA1 in the presence of lipid. **A)** Native 4–20% Tris-glycine PAGE gel, Native Tris-glycine buffer, detection by SyproRuby fluorescence total protein stain. **B)** Anti-YopD western blot of a native 4–20% Tris-glycine PAGE gel, Native Tris-glycine buffer, detection by fluorescence of rhodamine conjugated secondary antibody. The lanes are represented as follows: **1)** YopB with Δ49ApoA1; **2)** YopB/D with Δ49ApoA1; **3)** YopD with Δ49ApoA1; **4)** Δ49ApoA1 only (empty-NLP); **NM**) Protein mass standard (kDa) NativeMark (Invitrogen).

Cell-free expressed YopD is associated with NLPs. For an additional confirmation of the association of YopD with the NLPs as indicated by denatured and native electrophoresis and FCS, a western blot following native gel electrophoresis was performed (Fig 6). Using an anti-YopD antibody, YopD was detected in the characteristic NLP bands of samples co-expressing YopD and NLP forming Δ49A1, as well as samples co-expressed both YopB and YopD with the NLP forming Δ49A1 (Fig 6). No antibody interaction was detected in control samples of "empty"-NLPs or the YopB-NLPs (Fig 6B, lanes 1 and 4). Detection was only seen in the characteristic NLP bands on the native gel, where YopD was produced in the cell-free reaction (Fig 6A). In addition, no lower molecular weight bands were seen that would correspond to free protein, confirming that detectable YopD was associated with the NLPs despite the lack of height difference as determined by AFM. From our combined observations of SDS-PAGE and native western blot, we conclude that YopD was inserted in the NLPs.

Cell-free expressed YopB is associated with YopD. In order to confirm the association of YopB and YopD in NLPs, co-immunoprecipitation was performed on the YopD-NLPs and YopB/D-NLPs as well as lipid vesicle preparations of the two proteins with appropriate controls. YopD antibodies coupled to protein G-coated magnetic beads were used to capture co-expression products containing YopD. In the samples containing both Yop B and YopD, immunoprecipitation resulted in the co-precipitation of YopB (S1 Fig). Vesicle samples containing YopB did not bind to the antibody to a significant degree (S1 Fig, lane 4). YopD also appeared to be associated with the Δ49A1 NLP component. However, due to background binding of the “empty”-NLPs containing only the Δ49A1 and lipids, AFM and Anti-YopD Native PAGE western blots were needed to confirm the association of Yop proteins with the NLP. Importantly, these results indicate that YopB formed a complex with YopD, since it was precipitated with YopD. The control lipid vesicles with YopB alone showed a trace amount of non-specific binding. In the presence of YopD, YopB precipitation was significantly increased. These results, in addition to the stabilization and solubilization effect observed when expressing YopB in the presence of YopD (Fig 1), support complex formation between the YopB and YopD.

## Conclusion and Discussion

Results show that purification of YopB increased in the presence of YopD expression in cell-free extracts (Fig 1). Addition of lipids to the cell-free reactions enhanced the expression of YopB and/or YopD, but decreased the solubility (Fig 2). Soluble, stabilized expression of both YopB and YopD was dramatically increased using the co-expression NLP method (Fig 2, Table 1), which involves the cell-free expression of the Yop proteins in conjunction with the co-expression of NLP forming apolipoprotein and additive lipid vesicles. The resulting soluble YopB- and/or YopD-NLPs showed characteristic NLP migration on native gel (Fig 3). YopB and YopB/D containing NLPs were distinguishable from “empty”-NLPs using AFM height measurements (Fig 4) as previously described [17, 23–26]. FCS showed association of YopB and YopD with the NLPs (Fig 5) in solution without immobilization on a surface. A YopD native western blot confirmed the association of YopD with the characteristic NLP band (Fig 6). Co-immunoprecipitation confirmed the association of YopB and YopD in NLPs and vesicle preparations (S1 Fig). Inclusion of YopB and/or YopD in this native-like NLP bilayer likely mimics the structures of these proteins when incorporated into the host membrane as part of the T3SS. Moreover, NLPs provide a platform for the discovery of novel protein-protein interactions between the translocon complex and other T3SS injectisome proteins.

We have shown that our previously reported cell-free co-expression methods for producing membrane proteins in NLPs can produce components of the Yop membrane-protein translocon as an interacting complex, circumventing other less successful recombinant cellular expression approaches for producing these proteins. Cell-free protein yields are sufficient for both biochemical and biophysical assays, helping to resolve questions regarding complex formation and stoichiometry. We have characterized the production of YopB and/or YopD-NLPs using biophysical methods and show cell-free expression of YopB and YopD in NLPs is enhanced over the use of liposomes alone. Future interaction studies of the YopB/D translocon complex embedded in a membrane with effector proteins such as LcrV may elucidate the poorly understood host contact mechanism that allows this pathogen to evade the host defenses.

The ability to produce YopB and YopD in a more native-like membrane environment may prove valuable for plague vaccine development and may provide insight into the roles of YopB and YopD in effector protein translocation. Previous studies have shown that only partial protection from *Y*. *pestis* is acquired upon exposure to translocon components [13, 31–32], but have also suggested that these proteins are good candidates as protective antigens. However, the previous study that used recombinant cell methods failed to produce a sufficient quantity for large vaccination studies and did not provide the translocon proteins in their membrane-bound form. It will also be worthwhile to determine if the native-like presentation in the NLP construct provides enhanced immune stimulation. Moreover, current vaccine strategies using *Yersinia* V antigen (LcrV) and F1 capsule protein have some drawbacks as the F1 protein is not protective against F1^**-**^ strains [32], while naturally occurring or engineered polymorphism of LcrV may evade the protective effect provided by this antigen [33, 34]. YopB and YopD on the other hand possess a high degree of sequence homology to other near neighbor *Yersinia* species [19, 21]. Recombinant *Y*. *pseudotuberculosis* YopB and YopD have previously been reported to interact when over expressed. [13, 14] These same constructs have been shown to be protective against F1^-^ *Y*. *pestis*, offering cross-protective immunity in mice. The enhanced immunity in mice is most likely due to the high degree of sequence homology in YopB and YopD proteins [13, 21]. This current approach may enable larger-scale animal challenge studies which were not possible using previous approaches [13].

In conclusion, this is the first demonstration of a method producing a YopB/D complex in a single reaction using cell-free lysates combined with NLPs. These data show a viable method for making proteins for investigating their biochemistry without the noise from heterogeneous expression systems. The future goals of this research will be to scale reactions for structural studies and vaccine development.

## Materials and Methods

### Plasmids

The truncated form of Apo A1 (Δ1–49) or Δ49A1 was cloned as previously described in pIVEX2.4d using NdeI and SmaI restriction sites (17). This vector also contains a His-tag for nickel affinity purification. The YopB, YopD sequences from *Yesinia pestis* CO92 were cloned as described previously [35]. Briefly the gene of interest were PCR amplified with primers containing restriction sites for *Nde*I and *BamH*I and cloned into a modified pETBlue (Novagen) C-terminal His6 tagged system. YopB was subsequently sub-cloned directionally into the His-tagged pIVEX 2.4b vector (Roche).

### Cell-free Reactions

Small-scale reactions (100 uL) were carried out in triplicate using Life Technologies Expressway Maxi kit or Roche’s RTS 100 *E*. *coli* HY kit. In Brief, lyophilized reaction components (Lysate, Reaction Mix, Amino Acid Mix, and Methionine) are dissolved in reconstitution buffer and combined as specified by the manufacturer. To the reactions 1 μL FluoroTect™ Green_Lys_ (Promega) was added; which allows for the fluorescent labeling and detection of proteins synthesized during the cell-free translation. For small scale (100 μL) co-expression of YopB/D-NLPs the plasmid DNA was added in the following amounts, 0.5 μg of YopB, 1.0 μg YopD, and 0.1 μg Δ49A1 DNA. Plasmid amounts for co-expression of YopB-NLPs and YopD-NLPs were as follows: 0.5 μg of YopB and 0.1 μg of Δ49A1 DNAs; and 1.0 μg YopD and 0.1 μg of Δ49A1 DNAs, respectively. “Empty”-NLPs (no co-expressed Yop protein) were prepared using 1.0 μg of Δ49A1 DNA. To the lysate/DNA mixture DMPC vesicles (2 mg/mL) were added, see below. The reactions were incubated at 30°C, 900 rpm for 18 hrs in an Eppendorf thermomixer. Vesicle preparations were prepared similarly omitting the NLP-forming Δ49A1 DNA. Yop Protein alone samples were prepared as above omitting both the DMPC lipid component as well as the NLP-forming Δ49A1 DNA.

Preparative scale reactions (1 mL) were carried out using the Life Technologies Expressway Maxi kit or Roche’s RTS 500 ProteoMaster Kit. For co-expression of YopB/D-NLPs the plasmid DNA was added in the following amounts, 10 μg of YopD of 5 μg of YopB and 1.0 μg of Δ49A1. Plasmid amounts for YopB-NLPs and YopD-NLPs were as follows: 10 μg of YopB and 10 μg of Δ49A1 DNAs; and 10 μg YopD and 1 μg of Δ49A1 DNAs, respectively. “Empty”-NLPs (no co-expressed Yop protein) were prepared using 10 μg of Δ49A1 DNA. To the lysate/DNA mixture DMPC vesicles (2 mg/mL) were added, see below. The reactions were incubated at 30°C for 16 hrs. 40 mins. at 900 rpm, in a Roche ProteoMaster thermomixer.

### Lipid preparation

Small unilamellar vesicles of DMPC (liposomes) were prepared by probe sonicating a 68 mg/mL aqueous solution of DMPC until optical clarity is achieved; typically 15 min is sufficient. A 2 min. centrifugation step at 13700 RCF removed any metal contamination from the probe tip. The individual lipid component was added to the cell-free reaction at a concentration of 2 mg/mL.

### Affinity purification of NLP complexes

Immobilized metal affinity chromatography was used to isolate the protein complexes of interest (truncated 49A1 and YopB/D) from the cell-free reaction mixture based on the affinity of the N-terminal poly-His tag. The soluble fraction was separated from precipitated protein by centrifugation for 5 min at 18K RCF at 4°C. The soluble fraction was mixed at 4°C for 1 hr. with 1 mL of Probond Ni-NTA resin (Invitrogen Corp.) pre-equilibrated with PBS (50 mM Na_2_HPO_4_, 300 mM NaCl, pH 8.0) under native conditions in a 5 mL capped column. The column was washed with increasing concentrations of imidazole 5, 10 and 20 mM in the for mentioned PBS buffer. Two column volumes (CV) of each wash buffer were used for a total of 6 CVs of washing. The His-tagged proteins of interest were eluted in six 1mL fractions of 400 mM imidazole, PBS buffer. All elutions were combined, concentrated and buffer exchanged into TBS using a 100K MWCO molecular weight sieve filters (Vivascience) to a volume of ~200 uL.

### SDS PAGE Analysis

For small scale reactions, a 1 μL aliquot of the total (**T**) cell-free reaction, soluble (**S**) fraction and resuspended pellet (I) were diluted with 1x LDS Sample buffer with reducing agents (Invitrogen), heat denatured and loaded on to a 4–12% gradient pre-made Bis-Tris gel (Invitrogen) along with the molecular weight standard SeeBlue plus2 (Invitrogen). The running buffer was 1X MES-SDS (Invitrogen). Samples were electrophoresed for 38 minutes at 200V. Gels were imaged using the green laser (532 nm) of a Typhoon 9410 (GE Healthcare) with a 526 nm bandpass 30 filter for the detection of the produced Yop proteins and/or apolipoprotein with incorporated fluorescently labeled lysine (FluoroTect™ GreenLys in vitro Translation Labeling System, Promega), gels were subsequently stained with coomassie brilliant blue or SYPRO Ruby Protein Gel Stain (Bio-Rad) (Data not shown).

### Native PAGE Analysis

Equal mass aliquots of NLP samples (1.5 μg) were diluted with 2x native gel sample buffer (Invitrogen) and loaded onto 4–12% gradient pre-made Tris-glycine gels (Invitrogen). Samples were electrophoresed for 2 hrs. at a constant 125 V. After electrophoresis, gels were incubated with SYPRO Ruby protein gel stain (Bio-Rad) for 2 hours and then de-stained using 10% Methanol, 7% Acetic acid. Following a brief wash with ddH_2_O, gels were imaged using the green laser (532 nm) of a Typhoon 9410 (GE Healthcare) with a 610 nm bandpass 30 filter. Molecular weights were determined by comparing migration vs. log molecular weight of standard proteins found in the NativeMark standard (Invitrogen).

### Native Immunoblotting

Equal amounts of NLP samples (1.25 μg) were diluted with 2x native gel sample buffer (Invitrogen) and loaded onto duplicate 4–20% gradient pre-made Tris-glycine gels (Invitrogen). Samples were electrophoresed for 140 min. at a constant 125 V. One gel was stained and scanned for total protein using SYPRO Ruby protein gel stain (Bio-Rad) as above. The second gel was transferred to a PVDF membrane (Invitrogen) for 100 V, 60 min. The native blot was blocked for 1 ½ hours in blotto (5% powdered milk in 1X PBS, Gibco), the blot was incubated with primary anti-YopD antibody (rabbit) in at a 1:500 dilution in blotto over night at 4°C with mixing. The blot was washed 3X for a total of 30 min. in 1X PBS with 0.05% tween-20. A secondary fluorescent antibody was used for detection in the following concentrations, rhodamine conjugated goat-anti-mouse IgG 1:500 in blotto (Upstate), for 3 hours at 4°C. The blot was washed as above and imaged using the green laser (532 nm) of a Typhoon 9410 (GE Healthcare) with a 555 nm band pass 30 filter.

### Co-Immunoprecipitation

Protein G magnetic beads (Active Motif, Carlsbad, CA) were coupled to 20 μL anti-YopD antibodies (non-purified rabbit sera, generously provided by Dr. Gregory Plano) in 250 μL 1% BSA in 1X PBS, pH 7.4 (Gibco) for 1 hr. at 4°C, with mixing. The coupled beads were collected and resuspended in the above BSA/PBS buffer. Soluble cell-free extracts with co-expressed Yop-NLPs and "empty"-NLPs (20 uL); and crude cell-free extracts with expressed Yop proteins (20 μL) in the presence of lipid (vesicle preparations) were added to the anti-YopD Protein G magnetic resuspended beads. The mixture was incubated at 4°C for 4 ½ hrs. with mixing. The beads were washed with 4–500 μL aliquots of 1X PBS, pH 7.4. Collected beads were then re-suspended in 25 μL 2X LDS and heat denatured. Samples (25 μL) were loaded on a 4–12% Bis-Tris gel and electrophoresed for 38 min. at 200V. The resulting gel was imaged for the incorporated Fluorotect green-Lys (Promega) using the green laser (532 nm) of a Typhoon 9410 (GE Healthcare) with a 526 nm bandpass filter.

### Atomic Force Microscopy of Yop-NLPs

NLPs were imaged using and Asylum MFP-3D-CF atomic force microscope. Images were captured in tapping mode with minimal contact force and scan rates of 1 Hz. Asylum software was used for cross-sectional analysis to measure NLP height and diameter. For experimental analysis, the heights and diameters of NLPs assembled with different compositions (i.e. with and without YopB and YopD) were measured using the Asylum software. Two-tailed student T-tests were run to compare both the height and diameter of the different NLP populations. A p-value of <0.01 was considered significant.

### Fluorescence Correlation Spectroscopy of Yop-NLPs

NLPs were measured using a MicroTime 200 single molecule fluorescence lifetime measurement system (PicoQuant). Proteins labelled with Bodipy^®^-FL dye (for "empty" NLPs) and labelled with GFP (for YopD-NLPs or YopB/D-NLPs) were excited at 470 nm. The collected emission was filtered using a 520 ± 20 nm bandpass filter. Lipids labelled with Texas Red were excited at 470 nm. The collected emission was filtered using a 685 ± 35 nm bandpass filter. The single molecule fluorescence time traces were taken for ~3 minutes. All measurements were performed and analysed using the SymPhoTime Software (PicoQuant). Data were further analyzed using IGOR Pro 6 and OriginPro 8.

## Supporting Information

S1 Fig Co-immunoprecipitation of YopD confirms the association of YopB and YopD. Cell-free expressed YopB and /or YopD co-expressed with Δ49A1in the presence of lipid (lanes 1–3, Grey arrow indicates YopB, Black arrow indicates YopD, White arrow indicates Δ49A1 protein); We also compared expressed YopB and/or YopD in the presense of lipid vesicles (3–6) After expression all samples were immunoprecipitated with α-YopD antibody. Some level of Δ49A1 was seen as background due to binding to the G-protein beads used in the assay. Protein detection was accomplished by incorporation of fluortect (Promega) green-Lys lable. **A)** Native 4–12% Bis-Tris NuPAGE gel, Mes-SDS buffer, 200V, 38 min. The lanes are represented as follows: **M**) Protein mass standard (KDa) SeeBlue Plus2 (Invitrogen). **1)** YopB/D with Δ49A; **2)** YopD with Δ49ApoA1; **3)** Δ49ApoA1only (empty-NLP); **4**) YopB lipid vesicles; **5)** YopB/D lipid vesicles; **6)** YopD lipid vesicles.
(TIF)

## Abbreviations

Yop: *Yersinia* outer protein
T3SS: Type III secretion system
NLP: nanolipoprotein particle
rHDL: reconstituted high density lipoprotein
MP: membrane protein
DMPC: dimyristoyl-phosphatidylcholine
Δ49ApoA1: apolipoprotein A1 with amino acids 1–49 deleted

## References

[1] Plano GV, Day JB, Ferracci F. Type III export: new uses for an old pathway. Mol Microbiol. 2001 4;40(2):284–93. . Epub 2001/04/20. eng. 11309112 10.1046/j.1365-2958.2001.02354.x

[2] Brubaker RR. Interleukin-10 and inhibition of innate immunity to Yersiniae: roles of Yops and LcrV (V antigen). Infect Immun. 2003 7;71(7):3673–81. 12819047 10.1128/IAI.71.7.3673-3681.2003 PMC162007

[3] Kubori T, Matsushima Y, Nakamura D, Uralil J, Lara-Tejero M, Sukhan A, et al Supramolecular structure of the Salmonella typhimurium type III protein secretion system. Science. 1998 4 24;280(5363):602–5. . Epub 1998/05/09. eng. 9554854 10.1126/science.280.5363.602

[4] Holmstrom A, Olsson J, Cherepanov P, Maier E, Nordfelth R, Pettersson J, et al LcrV is a channel size-determining component of the Yop effector translocon of Yersinia. Mol Microbiol. 2001 2;39(3):620–32. . 11169103 10.1046/j.1365-2958.2001.02259.x

[5] Scherer CA, Cooper E, Miller SI. The Salmonella type III secretion translocon protein SspC is inserted into the epithelial cell plasma membrane upon infection. Mol Microbiol. 2000 9;37(5):1133–45. . 10972831 10.1046/j.1365-2958.2000.02066.x

[6] Cornelis GR. The type III secretion injectisome. Nature reviews Microbiology. 2006 11;4(11):811–25. . 17041629 10.1038/nrmicro1526

[7] Goure J, Broz P, Attree O, Cornelis GR, Attree I. Protective anti-V antibodies inhibit Pseudomonas and Yersinia translocon assembly within host membranes. J Infect Dis. 2005 7 15;192(2):218–25. . 15962216 10.1086/430932

[8] Mueller CA, Broz P, Muller SA, Ringler P, Erne-Brand F, Sorg I, et al The V-antigen of Yersinia forms a distinct structure at the tip of injectisome needles. Science. 2005 10 28;310(5748):674–6. . 16254184 10.1126/science.1118476

[9] Mota LJ. Type III secretion gets an LcrV tip. Trends Microbiol. 2006 5;14(5):197–200. 16564172 10.1016/j.tim.2006.02.010

[10] Edqvist PJ, Aili M, Liu J, Francis MS. Minimal YopB and YopD translocator secretion by Yersinia is sufficient for Yop-effector delivery into target cells. Microbes Infect. 2007 2;9(2):224–33. . 17223369 10.1016/j.micinf.2006.11.010

[11] Benner GE, Andrews GP, Byrne WR, Strachan SD, Sample AK, Heath DG, et al Immune response to Yersinia outer proteins and other Yersinia pestis antigens after experimental plague infection in mice. Infect Immun. 1999 4;67(4):1922–8. . Epub 1999/03/20. eng. 10085037 10.1128/iai.67.4.1922-1928.1999 PMC96547

[12] Olsson J, Edqvist PJ, Broms JE, Forsberg A, Wolf-Watz H, Francis MS. The YopD translocator of Yersinia pseudotuberculosis is a multifunctional protein comprised of discrete domains. J Bacteriol. 2004 7;186(13):4110–23. . 15205412 10.1128/JB.186.13.4110-4123.2004 PMC421591

[13] Ivanov MI, Noel BL, Rampersaud R, Mena P, Benach JL, Bliska JB. Vaccination of mice with a Yop translocon complex elicits antibodies that are protective against infection with F1- Yersinia pestis. Infect Immun. 2008 11;76(11):5181–90. 10.1128/IAI.00189-08 18765742 PMC2573372

[14] Ryndak MB, Chung H, London E, Bliska JB. Role of predicted transmembrane domains for type III translocation, pore formation, and signaling by the Yersinia pseudotuberculosis YopB protein. Infect Immun. 2005 4;73(4):2433–43. . 15784589 10.1128/IAI.73.4.2433-2443.2005 PMC1087397

[15] Klammt C, Schwarz D, Lohr F, Schneider B, Dotsch V, Bernhard F. Cell-free expression as an emerging technique for the large scale production of integral membrane protein. Febs J. 2006 9;273(18):4141–53. . 16930130 10.1111/j.1742-4658.2006.05432.x

[16] Chromy BA, Arroyo E, Blanchette CD, Bench G, Benner H, Cappuccio JA, et al Different apolipoproteins impact nanolipoprotein particle formation. J Am Chem Soc. 2007;129(46):14348–54. 17963384 10.1021/ja074753y

[17] Cappuccio JA, Blanchette CD, Sulchek TA, Arroyo ES, Kralj JM, Hinz AK, et al Cell-free co-expression of functional membrane proteins and apolipoprotein forming soluble nanolipoprotein particles. Mol Cell Proteomics. 2008 7 4 . 18603642 10.1074/mcp.M800191-MCP200 PMC2577204

[18] Ly S, Bourguet F, Fischer NO, Lau EY, Coleman MA, Laurence TA. Quantifying interactions of a membrane protein embedded in a lipid nanodisc using fluorescence correlation spectroscopy. Biophysical journal. 2014 1 21;106(2):L05–8. Pubmed Central PMCID: 3907250. 10.1016/j.bpj.2013.12.014 24461026 PMC3907250

[19] Hakansson S, Bergman T, Vanooteghem JC, Cornelis G, Wolf-Watz H. YopB and YopD constitute a novel class of Yersinia Yop proteins. Infect Immun. 1993 1;61(1):71–80. . 8418066 10.1128/iai.61.1.71-80.1993 PMC302689

[20] Segelke BW, Schafer J, Coleman MA, Lekin TP, Toppani D, Skowronek KJ, et al Laboratory scale structural genomics. J Struct Funct Genomics. 2004;5(1–2):147–57. . 15263853 10.1023/B:JSFG.0000029193.82120.d1

[21] Chain PS, Carniel E, Larimer FW, Lamerdin J, Stoutland PO, Regala WM, et al Insights into the evolution of Yersinia pestis through whole-genome comparison with Yersinia pseudotuberculosis. Proc Natl Acad Sci U S A. 2004 9 21;101(38):13826–31. . Epub 2004/09/11. eng. 15358858 10.1073/pnas.0404012101 PMC518763

[22] Sawasaki T, Hasegawa Y, Tsuchimochi M, Kamura N, Ogasawara T, Kuroita T, et al A bilayer cell-free protein synthesis system for high-throughput screening of gene products. FEBS Lett. 2002 3 6;514(1):102–5. . 11904190 10.1016/s0014-5793(02)02329-3

[23] Blanchette CD, Cappuccio JA, Kuhn EA, Segelke BW, Benner WH, Chromy BA, et al Atomic force microscopy differentiates discrete size distributions between membrane protein containing and empty nanolipoprotein particles. Biochim Biophys Acta. 2009 3;1788(3):724–31. 10.1016/j.bbamem.2008.11.019 19109924

[24] Cappuccio JA, Hinz AK, Kuhn EA, Fletcher JE, Arroyo ES, Henderson PT, et al Cell-free expression for nanolipoprotein particles: building a high-throughput membrane protein solubility platform. Methods Mol Biol. 2009;498:273–96. 10.1007/978-1-59745-196-3_18 18988032

[25] Katzen F, Fletcher JE, Yang JP, Kang D, Peterson TC, Cappuccio JA, et al Insertion of membrane proteins into discoidal membranes using a cell-free protein expression approach. J Proteome Res. 2008 8;7(8):3535–42. 10.1021/pr800265f 18557639

[26] Baker SE, Hopkins RC, Blanchette CD, Walsworth VL, Sumbad R, Fischer NO, et al Hydrogen production by a hyperthermophilic membrane-bound hydrogenase in water-soluble nanolipoprotein particles. J Am Chem Soc. 2009 6 10;131(22):7508–9. Epub 2009/05/20. eng. 10.1021/ja809251f 19449869

[27] Francis MS, Wolf-Watz H. YopD of Yersinia pseudotuberculosis is translocated into the cytosol of HeLa epithelial cells: evidence of a structural domain necessary for translocation. Mol Microbiol. 1998 8;29(3):799–813. . 9723919 10.1046/j.1365-2958.1998.00973.x

[28] Tengel T, Sethson I, Francis MS. Conformational analysis by CD and NMR spectroscopy of a peptide encompassing the amphipathic domain of YopD from Yersinia. Eur J Biochem. 2002 8;269(15):3659–68. . 12153562 10.1046/j.1432-1033.2002.03051.x

[29] Tardy F, Homble F, Neyt C, Wattiez R, Cornelis GR, Ruysschaert JM, et al Yersinia enterocolitica type III secretion-translocation system: channel formation by secreted Yops. Embo J. 1999 12 1;18(23):6793–9. . 10581252 10.1093/emboj/18.23.6793 PMC1171741

[30] Gao T, Blanchette CD, He W, Bourguet F, Ly S, Katzen F, et al Characterizing diffusion dynamics of a membrane protein associated with nanolipoproteins using fluorescence correlation spectroscopy. Protein science: a publication of the Protein Society. 2011 2;20(2):437–47. . Pubmed Central PMCID: 3048428. 21280134 10.1002/pro.577 PMC3048428

[31] Andrews GP, Strachan ST, Benner GE, Sample AK, Anderson GW Jr., Adamovicz JJ, et al Protective efficacy of recombinant Yersinia outer proteins against bubonic plague caused by encapsulated and nonencapsulated Yersinia pestis. Infect Immun. 1999 3;67(3):1533–7. . 10024607 10.1128/iai.67.3.1533-1537.1999 PMC96493

[32] Smiley ST. Immune defense against pneumonic plague. Immunol Rev. 2008 10;225:256–71. 10.1111/j.1600-065X.2008.00674.x 18837787 PMC2804960

[33] Motin VL, Pokrovskaya MS, Telepnev MV, Kutyrev VV, Vidyaeva NA, Filippov AA, Smirnov GB.The difference in the lcrV sequences between Y. pestis and Y. pseudotuberculosis and its application for characterization of Y. pseudotuberculosis strains. Microb Pathog. 1992 3;12(3):165–75. 1614327 10.1016/0882-4010(92)90050-x

[34] Anisimov AP, Dentovskaya SV, Panfertsev EA, Svetoch TE, Kopylov PKh, Segelke BW, Zemla A, Telepnev MV, Motin VL. Amino acid and structural variability of Yersinia pestis LcrV protein. Infect Genet Evol. 2010 1;10(1):137–45. 10.1016/j.meegid.2009.10.003 19835996 PMC2818281

[35] Raab R, Swietnicki W. Yersinia pestis YopD 150–287 fragment is partially unfolded in the native state. Protein expression and purification. 2008 3;58(1):53–60. . 18160307 10.1016/j.pep.2007.11.001

