# Supplementary figures and images for: Expression and Association of the Yersinia pestis Translocon Proteins, YopB and YopD, Are Facilitated by Nanolipoprotein Particles
Source: PLoS One. 2016 Mar 25;11(3):e0150166. doi: 10.1371/journal.pone.0150166 (PMC4807764; doi:10.1371/journal.pone.0150166)

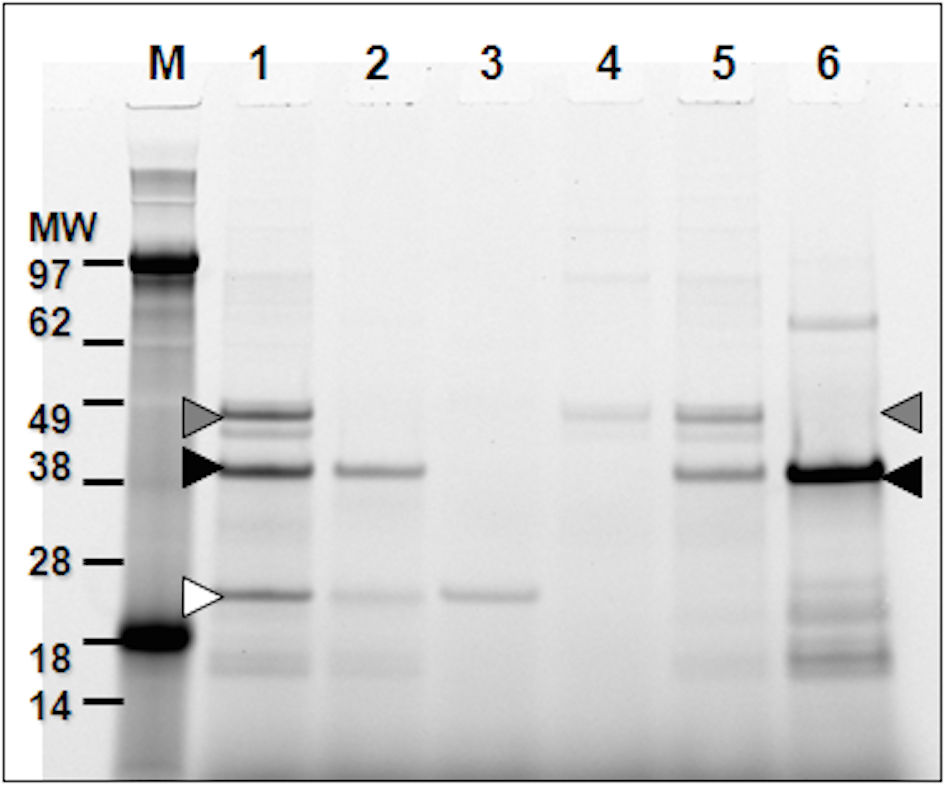

Supplement: S1 Fig — Cell-free expressed YopB and /or YopD co-expressed with Δ49A1in the presence of lipid (lanes 1–3, Grey arrow indicates YopB, Black arrow indicates YopD, White arrow indicates Δ49A1 protein); We also compared expressed YopB and/or YopD in the presense of lipid vesicles (3–6) After expression all samples were immunoprecipitated with α-YopD antibody. Some level of Δ49A1 was seen as background due to binding to the G-protein beads used in the assay. Protein detection was accomplished by incorporation of fluortect (Promega) green-Lys lable. A) Native 4–12% Bis-Tris NuPAGE gel, Mes-SDS buffer, 200V, 38 min. The lanes are represented as follows: M) Protein mass standard (KDa) SeeBlue Plus2 (Invitrogen). 1) YopB/D with Δ49A; 2) YopD with Δ49ApoA1; 3) Δ49ApoA1only (empty-NLP); 4) YopB lipid vesicles; 5) YopB/D lipid vesicles; 6) YopD lipid vesicles. (TIF) [file pone.0150166.s001.tif]
